# Apparent Thixotropic Properties of Saline/Glycerol Drops with Biotinylated Antibodies on Streptavidin-Coated Glass Slides: Implications for Bacterial Capture on Antibody Microarrays

**DOI:** 10.3390/s90200995

**Published:** 2009-02-16

**Authors:** David M. Albin, Andrew G. Gehring, Sue A. Reed, Shu-I Tu

**Affiliations:** Microbial Biophysics and Residue Chemistry Research Unit, USDA-ARS, Eastern Regional Research Center, Wyndmoor, PA 19038, USA; E-Mails: david_m_albin@yahoo.com; sue.reed@ars.usda.gov; shui.tu@ars.usda.gov

**Keywords:** Fluorescence immunoassay, Antibody microarray, Bacteria, Print Buffer

## Abstract

The thixotropic-like properties of saline/glycerol drops, containing biotinylated capture antibodies, on streptavidin-coated glass slides have been investigated, along with their implications for bacterial detection in a fluorescent microarray immunoassay. The thixotropic-like nature of 60:40 saline-glycerol semisolid droplets (with differing amounts of antibodies) was observed when bacteria were captured, and their presence detected using a fluorescently-labeled antibody. Semisolid, gel-like drops of biotinylated capture antibody became liquefied and moved, and then returned to semisolid state, during the normal immunoassay procedures for bacterial capture and detection. Streaking patterns were observed that indicated thixotropic-like characteristics, and this appeared to have allowed excess biotinylated capture antibody to participate in bacterial capture and detection. When developing a microarray for bacterial detection, this must be considered for optimization. For example, with the appropriate concentration of antibody (in this study, 0.125 ng/nL), spots with increased diameter at the point of contact printing (and almost no streaking) were produced, resulting in a maximal signal. With capture antibody concentrations greater than 0.125 ng/nL, the excess biotinylated capture antibody (i.e., that which was residing in the three-dimensional, semisolid droplet space above the surface) was utilized to capture more bacteria. Similarly, when the immunoassay was performed within a hydrophobic barrier (i.e., without a coverslip), brighter spots with increased signal were observed. In addition, when higher concentrations of cells (∼10^8^ cells/mL) were available for capture, the importance of unbound capture antibody in the semisolid droplets became apparent because washing off the excess, unbound biotinylated capture antibody before the immunoassay was performed reduced the signal intensity by nearly 50%. This reduction in signal was not observed with lower concentrations of cells (∼10^6^ cells/mL). With increased volumes of capture antibody, abnormal spots were visualized, along with decreased signal intensity, after bacterial detection, indicating that the increased droplet volume detrimentally affected the immunoassay.

## 1. Introduction

The wetting properties (and droplet formation) of solutions on surfaces have long been an area of interest [1,2]. Currently, these characteristics are under study due to their importance in several technologies, including composites, printing, coatings, and oil recovery [3,4]. Liquid and colloidal solutions exhibit wetting and droplet formations to varying degrees, depending on their composition [1]. Many semisolid, gel-like solutions form droplets with poor wetting properties, and therefore, make limited contact with a surface. These solutions exhibit thixotropic-like characteristics, where the droplets are semisolid and gel-like until acted upon by an outside force, such as lateral shearing or shaking, after which they become liquefied [sol phase; 5]. When the force is removed, the semisolid character returns [5]. The thixotropic behavior of suspensions of biomolecules has also been examined [6]. More recently, attaching biomolecules (especially antibodies) to glass surfaces for immunosensor development has become an active area of research [7]. The optimal buffers, storage conditions, and other procedures to attach biomolecules to glass surfaces, such as microarrays, are beginning to be developed [8,9].

Microarrays are traditionally orthogonally-arrayed micron-diameter spots, at micron-spaced distances on microscope slides (typically referred to as substrates), which contain biomolecules that are chemically attached to the surface. To produce the spots, small droplets are applied to the surface using either robotic or manual printing techniques. Microarrays have been used extensively in the past 10 years, especially those containing nucleic acid sequences for gene expression studies [10]. More recently, microarrays containing protein have been developed and used to study protein-protein interactions [11]. Perhaps the most significant characteristic of microarrays, and the reason for their popularity, is their ability to contain thousands of spots per substrate, and therefore, simultaneously accommodate thousands of analyses with a single sample. Thus, in the past few years, efforts to produce microarray biosensors, which serve diagnostic purposes, have been undertaken [12-14]. In particular, combining the sandwich immunoassay with microarray format is a current area of interest [12,13,15].

In order to reduce stresses on immobilized antibodies, print buffers with various salts, surfactants, and stabilizers have been developed [9]. In an early protein microarray article [11], antibodies were reconstituted in phosphate-buffered saline (PBS) plus 40% glycerol, and a recent report [16] has indicated that PBS with 20% glycerin (glycerol) produced a superior microarray response signal relative to PBS alone. The authors speculated that glycerol served as a protein stabilizer by maintaining a hydrated state [16]. We recently developed an antibody microarray method for the capture and detection of *E. coli* O157:H7 [17]. It became apparent that the interactions of the biotinylated capture antibodies in PBS/glycerol spots with the streptavidin-coated glass substrate markedly affected the immunoassay, at least in terms of whole bacterial cell detection. Therefore, in this study, evidence for thixotropic-like properties of the glycerol-containing spots is presented, and the implications of these properties on bacterial capture and immunoassay results, within a protein microarray format, are examined.

## 2. Results and Discussion

In order to determine background fluorescent signals, the appropriate blank samples were analyzed. Immunoassays performed without bacteria, but treated with reporter antibody, generated fluorescent signals that were less than, or equal to, the localized background AFU (arbitrary fluorescence units; data not shown) measurements. Similarly, following bacterial capture by biotinylated capture antibodies, assays performed without reporter antibody also generated fluorescent signals that were less than, or equal to, the localized background AFU (arbitrary fluorescence units; data not shown) measurements.

### 2.1. Influence of Lateral Shearing on Biotinylated Antibodies in PBS/Glycerol Spots

**Figure 1.**
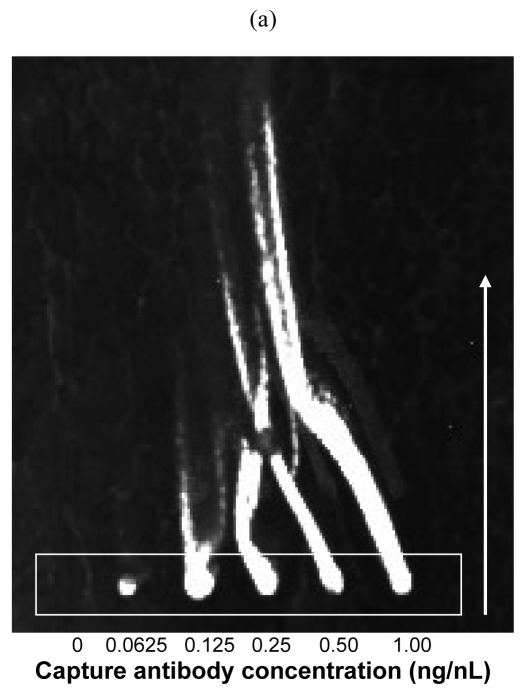

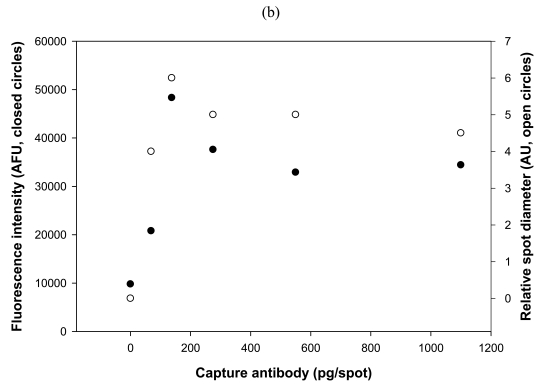
(a) Spread of differing concentrations of biotinylated anti-*E. coli* O157:H7 capture antibodies (white box indicates site of contact printing by microarray printer), in 135 μm diameter spots (1.1 nL) of 60% PBS:40% glycerol solution (v/v), across streptavidin-coated microscope slides following shearing force (100 μL PBS plus 1% BSA solution [wt/v] applied to one end of a microarray coverslip for blocking purposes; white arrow indicates directionality of flow). After 1 h incubation, the slide was used to capture and detect 1.6 × 10^9^ *E. coli* O157:H7 cells using a sandwich immunoassay. (b) Correlation between fluorescence intensity (shown in arbitrary fluorescence units, or AFU) and relative spot diameter (measured with a ruler and shown in arbitrary units, or AU), following completion of sandwich immunoassay, at the site of contact printing in Figure 1a.

The effect of a shearing force (associated with the blocking step), applied to serial dilutions of biotinylated capture antibody, on subsequent capture and detection of *E. coli* O157:H7 is shown in Figure 1(a). One hundred microliters of blocking solution (PBS plus 1% BSA) was applied to one end of a microarray cover slip, and the solution flowed across the surface via capillary action, applying a shearing force to the spots. Biotinylated capture antibodies in 60% PBS:40% glycerol were printed onto streptavidin-coated slides, and the shearing force affected the unbound capture antibodies in the semisolid droplets. The bacterial capture and detection procedures were then completed, and upon fluorescent slide scanning, the spots exhibited streaking that was dependent upon the concentration of biotinylated antibody [Figure 1(a)]. Thus, with approximately 0.125 ng/nL biotinylated capture antibody (or 137.5 pg per spot) and higher concentrations (printed with SMP4 pins; 1.1 nL delivery volume; 135 μm spot diameter), the capture antibody was in excess (i.e., the streptavidin binding sites at the slide surface were saturated with biotinylated antibodies) and spread over the slide. Therefore, a capture antibody concentration of about 0.1 ng/nL, printed with SMP4 pins, would produce a droplet that allowed maximal surface contact relative to the amount of capture antibody. Indeed, the concentration that resulted in the largest fluorescent response and the widest spot diameter (as measured with a ruler and expressed in arbitrary units, or AU) at the point of contact printing (and minor streaking) was 0.125 ng/nL [Figure 1(b)].

From the image shown in Figure 1(a), the thixotropic-like properties of PBS/glycerol drops can be observed. From capture antibody concentrations of 0.125 ng/nL and greater, the surface at the points of contact printing on the streptavidin-coated slides became saturated with biotinylated antibodies. Upon lateral shearing with the blocking solution, excess capture antibody that was occupying the three-dimensional space above the microarray slide was forced over the streptavidin-coated slide (sol-like phase was apparently generated). During the hour-long blocking time, the PBS/glycerol solution exhibited properties like thixotropy (the original semisolid, gel phase resumed), which allowed the unbound biotinylated capture antibodies to interact with, and bind to, the streptavidin surface. When bacteria were then presented for capture and detection, these capture antibodies could then participate in the immunoassay (i.e., they were not removed in the washing steps).

### 2.2. Effect of a Coverslip on Bacterial Capture and Detection

**Figure 2.**
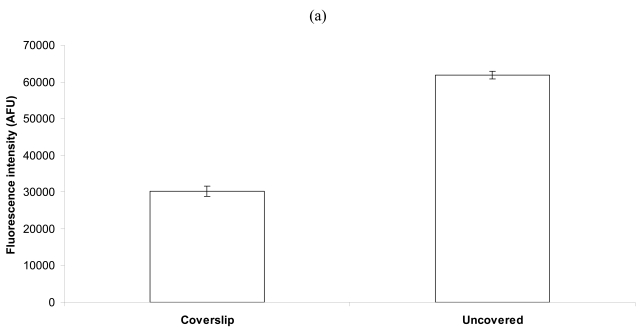

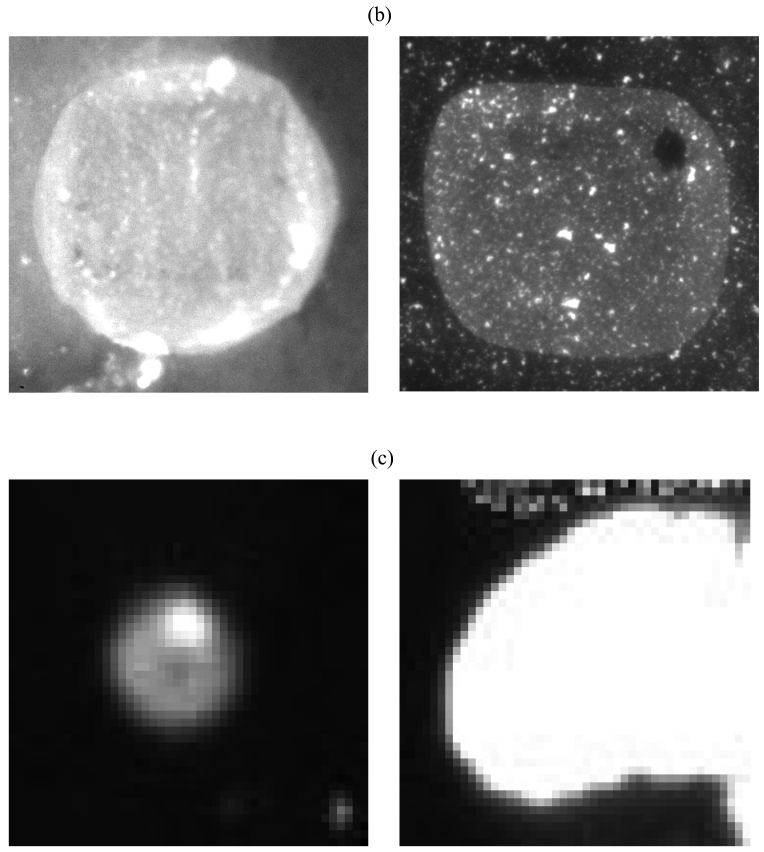
Bacterial capture as affected by the use of a coverslip. (a) Detection of 4.7 × 10^8^ cells/mL (with 1 ng capture antibody/nL spots) exhibited lower AFU (arbitrary fluorescence units, background corrected) values with the use of a coverslip versus a hydrophobic barrier. (b) Fluorescence microscopic images of covered (left) and uncovered (right) microarray spots (Triplicate 135 μm diameter spots per slide, each on 2 slides) following capture and detection of bacteria. The use of a coverslip produced more uniform, compact spots (and lower AFU measurements), while the hydrophobic barrier approach produced less uniform spots (and higher AFU measures**).** (c) With the array slide scanner, uncovered spots (right) had wider diameters, and were more intense, after capture and detection of bacteria. Note, the presented spots (scan images and fluorescent micrographs) are representative of day-to-day replicated experimental observations and are displayed at an identical scale for each left-right pair of images. In addition, the images in (b) are more tightly cropped relative to those in (c).

To incubate liquids on the microarray surface, a microarray cover slip can be used, or a hydrophobic barrier can be drawn around the spots. As shown in Figure 2(a), the use of coverslip resulted in less fluorescence intensity (approximately 50%) compared to a hydrophobic barrier. It should also be noted that the hydrophobic barrier occupied less surface area compared to the cover slip, while both were exposed to the same volume. Thus, with a cover slip, only about 8% as many bacterial cells came into contact with each spot relative to the hydrophobic barrier technique. Also, with a hydrophobic barrier, the bacterial cells can be added directly over the spots, while a cover slip requires that the cells be added at one end and flow across the slide via capillary action.

With the addition of solutions directly over the spots (versus lateral shearing flow under a cover slip), unbound biotinylated capture antibodies in the droplets were allowed to spread across the streptavidin-coated surface and attach to it. This can be observed in fluorescent micrographs [Figure 2(b); bright dots or clumps represent captured “bacterial objects”-comprised mainly of bacterial cells and cell fragments] and microarray laser scanner images [Figure 2(c)]. Therefore, as in Figure 1(a), the apparent thixotropy of PBS/glycerol drops allowed the droplets of capture antibodies to spread outward in all directions when liquid was applied from above. Note, the images in Figure 2(b) are more tightly cropped (closer in) and of higher resolution relative to those in Figure 2(c). Because of that, the initial arrayed spot(s) can be clearly distinguished [in Figure 2(b)] from the bacterial objects that bound to capture antibody within and adjacent to the initial spots. In addition, the differences in cropping, excitation dwell times (hence, differential quenching), and resolution between the right images in Figures 2(b) and 2(c) suppressed the intensity of fluorescence contributed by the bacterial objects and exclude the presence of the “comet tail” in the right-hand part of the spot. Furthermore, the differences in focusing (non-confocal fluorescence microscopy versus confocal laser scanning) resulted in the lack of some characteristic features in the left images in Figures 2(b) and 2(c).

### 2.3. Effect of Removal of Unbound Capture Antibody Prior to Bacterial Capture

**Figure 3.**
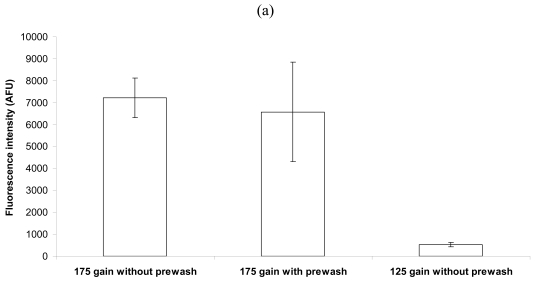

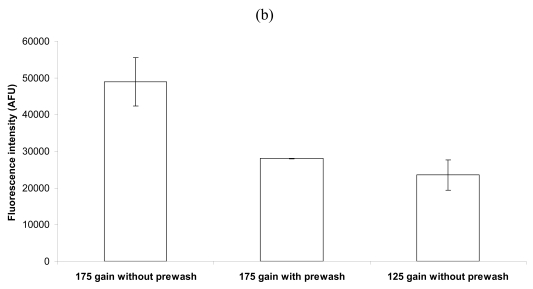
Effects due to scanning at 175 or 125 PMT gain, with or without slide washing before bacterial capture (Five 135 μm diameter, 1 ng/nL spots per slide, each on 2 slides, using a coverslip), at 4.2 × 10^6^ (a) or 4.2 × 10^8^ (b) cells/mL. (a) Slide washing before capture did not affect AFU (arbitrary fluorescence units, background corrected) when scanned at 175 gain. Lowering the gain to 125 reduced fluorescence intensity. (b) **A**t 10^8^ cells/mL, slide washing before capture reduced AFU values with 175 gain scanning, which was similar to the unwashed, 125 gain scanned slides.

After spotting capture antibody to the streptavidin-coated surface, the droplets were typically unaltered before the blocking solution was applied. Thus, at high concentrations, unbound capture antibody resided in the three-dimensional space over the surface [see Figure 1(a)]. When the slides were washed in PBS for 3 min, and spun dry, prior to blocking, a concentration dependence effect was observed [Figures 3(a)-(b)].

With a low concentration of bacterial cells [10^6^ cells/mL, Figure 3(a); close to lower limit of detection, see 17], washing the slides to remove unbound capture antibody before blocking and bacterial capture did not affect the fluorescence intensity. Also, when the photomultiplier tube gain (PMT, a measure of the intensity of the fluorescence laser scan) was reduced from 175 to 125 (and without removing the unbound capture antibody prior to blocking), the intensity was markedly reduced [Figure 3(a)]. With a high, saturation level of bacteria [10^8^ cells/mL, Figure 3(b); saturation concentration, see 17], washing the slides prior to blocking and bacterial capture reduced the fluorescence intensity by nearly 50%. In addition, when the slides were not washed before blocking and capture, and the PMT gain was reduced to 125, the intensity was similar to the response obtained with 175 gain and prewashing of slides [Figure 3(b)]. Therefore, with an abundance of cells for capture, the unbound capture antibodies in the droplets were important to produce a maximal signal. When few cells were available for capture, this was not a concern.

### 2.4. Effects Due to Spot Size

**Figure 4.**
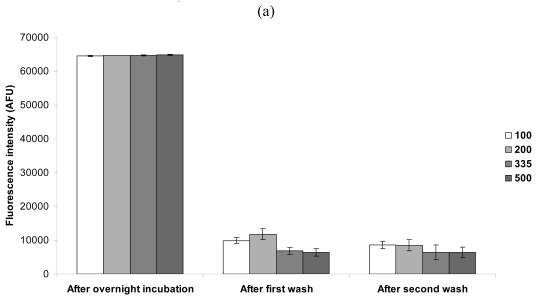

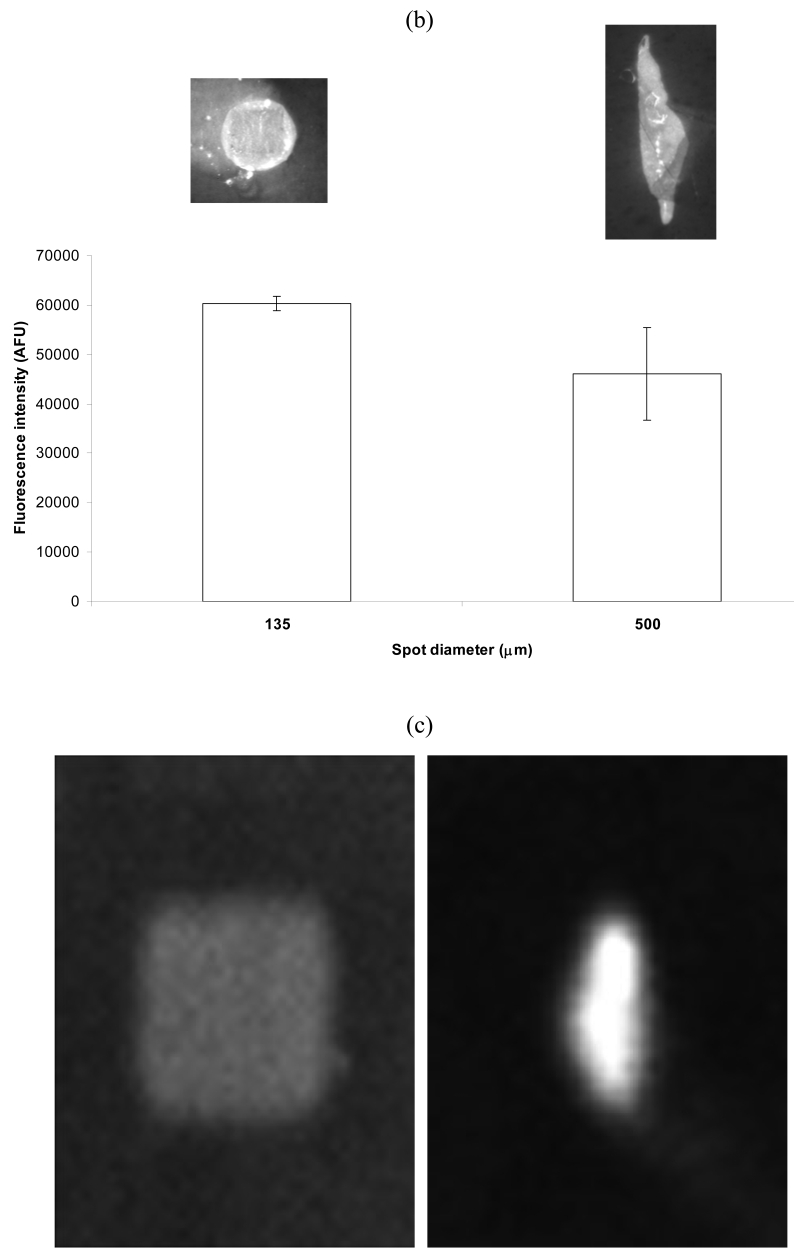
Effects due to spot size. (a) Biotinylated fluorescein was spotted at four spot diameters (duplicate spots of each size, micron diameters shown in legend, each on 3 slides), scanned, and washed twice with scanning after each wash. Washing reduced fluorescence intensity (AFU = arbitrary fluorescence units, background corrected) at all spot diameters. At 100 μm, AFU values were not reduced from the first wash to second wash. However, at 200 μm, AFU was reduced from the first to second wash. At 335 and 500 μm, washing twice versus once did not reduce AFU measurements, but these largest two spot diameters consistently exhibited lower AFU values after the washings compared with 100 and 200 μm diameters. (b) Bacterial cells (3.3 × 10^8^ cells/mL) exhibited increased signal with smaller, 135 μm spots, as opposed to larger, 500 μm spot diameters (duplicate spots of each size, each on 3 uncovered slides). Also, larger spots appeared to have altered spot morphology (fluorescent microscopic images shown above appropriate bars). (c) Five hundred micron spot images, produced with fluorescence laser scanner, showed expected spot morphology with biotinylated fluorescein (left image), and altered spot morphology after capture and detection of bacteria (right image). Note, the presented spots (scan images and fluorescent micrographs) are representative of day-to-day replicated experimental observations and are displayed at an identical scale for each left-right pair of images.

Considering the data presented thus far, it is apparent that excess, unbound biotinylated capture antibody resided in PBS/glycerol droplets, directly above the streptavidin-coated surface. In addition, during normal immunoassay conditions to capture and detect bacteria, the droplets exhibited thixotropic-like properties that affected the assay results. Thus, printing larger spot sizes (increased print volumes that will produce wider diameters), to evenly spread capture antibody over a wider spot, could produce wider, more uniform, and brighter spots. First, in order to determine if larger volumes/spot diameters will spread over the streptavidin surface, and bond evenly throughout the spot, biotinylated fluorescein was spotted at 4 diameters (100, 200, 335, and 500 μm). Following an overnight incubation, the slides were scanned and washed in PBS, and then rescanned to determine if print volume/spot diameter affected biotin-streptavidin bonding. As shown in Figure 4A, following overnight incubation, all spot diameters exhibited maximized fluorescence intensity that exceeded the scanner's upper threshold. Following a wash in PBS, fluorescence was reduced, especially with the large spot diameters (335 and 500 μm). After a second PBS wash, fluorescence intensity was similar at all spot diameters (although still somewhat lower with larger spot diameters), and was similar to the signal following the first wash, indicating that biotin was firmly bound to the streptavidin-coated surface.

When biotinylated capture antibodies were printed at two spot diameters (135 and 500 μm), and bacteria were captured and detected, a greater (and less variable) signal was generated with the 135 μm diameter spots (Figure 4B). In addition, fluorescent micrographs (Figure 4B) indicated that the 135 μm diameter spots exhibited normal, round spot morphology, while the 500 μm spots displayed abnormal, narrowed spot morphology.

Images of the 500 μm diameter spots produced with the fluorescent laser scanner are shown in Figure 4C. When biotinylated fluorescein was spotted (left image), a normal, round spot was visualized after two PBS washes. However, when biotinylated capture antibodies were spotted, and bacteria were captured and detected (right image), abnormal, narrowed spots were produced.

It is possible that the larger volumes of capture antibody solution that were printed onto the microarray slides did not distribute evenly, relative to biotinylated fluorescein solutions (possibly owing to 40% glycerol solutions). In fact, Alteraifi *et al.* [3] reported that glycerol drops spread incompletely on glass (relative to other drops). However, when biotinylated fluorescein was printed in this manner (40% glycerol solution), spots with expected morphology were produced at all spot sizes. Therefore, solution composition (biotinylated fluorescein versus biotinylated capture antibody) may markedly affect spreading rate on the glass surface used in the present study. In addition, the immunocomplex (i.e., capture antibody, captured bacteria, and reporter antibody) produced in this study was much larger (and perhaps less stable) than biotinylated fluorescein alone on the array slides. Indeed, Yang *et al.* [18], who developed an immuno-chip with 500 μm diameter spots for the capture of human leukocytes, reported amorphic spots after capture and detection. One study [19], using a flow cell microarray biosensor, reported that spot size had little effect on antigen-antibody association rate, as long as the spots were large enough to be visualized with the detector. Further research is necessary; however, under the experimental conditions used here, using smaller spot sizes (100 or 135 μm diameter) appeared to be superior to larger ones for capture and/or detection of bacteria.

## 3. Experimental Section

### 3.1. Materials

Materials used in this research included the following: biotinylated goat anti-*E. coli* O157:H7 antibody (used as capture antibody; 1 ng/nL, or diluted as indicated throughout), and fluorescein-labeled goat anti-*E. coli* O157:H7 antibody (used as reporter antibody; 0.5 ng/nL), from Kirkegaard & Perry Laboratories, Inc. (Gaithersburg, MD); biotinylated fluorescein conjugate (1 ng/nL) from Calbiochem-Novabiochem Corporation (San Diego, CA); biotin-labeled bovine albumin (1 μg/μL), streptavidin (0.2 μg/μL), PBS tablets, glycerol, Trizma^®^ base, and bovine serum albumin (BSA; fraction V) from Sigma-Aldrich (St. Louis, MO); Superfrost™ Gold electrostatically-coated microscope slides and LifterSlip™ m series microarray coverslips (22 mm × 50 mm) from Erie Scientific Company (Portsmouth, NH); PAP hydrophobic barrier pens from BioGenex Laboratories (San Ramon, CA); *E. coli* O157:H7 B1409 from Centers for Disease Control (Atlanta, GA); and EC medium from DIFCO Laboratories (Detroit, MI). Other chemicals were of reagent grade.

### 3.2. Apparatus

Solutions were printed onto microarray slides using the SpotBot^®^ Personal Microarray Robot (protein version; TeleChem International, Inc., Sunnyvale, CA). Fluorescent images of the microarray slides were produced with the Tecan LS400 laser slide scanner (Research Triangle Park, NC). Incubations with shaking were conducted with the innOva™ 4000 from New Brunswick Scientific (Edison, NJ). A Petroff-Hausser counting chamber from Thomas Scientific (Swedesboro, NJ) was used to enumerate bacterial cells. Washing of microarray slides was conducted with the Stain Train System (slide holders and washing jars) from MarketLab (Kentwood, MI). Microarray slides were coated with streptavidin using polypropylene Coplin jars and polystyrene slide washing jars from VWR (West Chester, PA).

### 3.3. Streptavidin-Coating of Microarray Slides

Streptavidin-coated slides were prepared as described on Dr. Andrew Flavell's website (The University of Dundee, Dundee, U.K.: http://www.personal.dundee.ac.uk/~ajflavel/TAM_protocol.htm; see Appendix 3). Briefly, five Superfrost Gold slides were placed in a siliconized polypropylene Coplin jar and incubated in 20 mL biotin-labeled bovine albumin solution (prepared in T50 buffer - 10 mM Trizma^®^, 50 mM NaCl, pH 8.0) for 45 min at room temperature (RT). Twenty milliliters covered approximately two-thirds of each slide, and the slides were periodically mixed. After two washes (10 min each) in T50 buffer in a polystyrene slide washing jar, the slides were incubated in another siliconized polypropylene Coplin jar in streptavidin solution (in T50 buffer) for 10 min at RT. After two washes (10 min each) in T50 buffer, the slides were briefly rinsed in distilled, deionized H_2_O and allowed to dry in a fume hood for approximately 1 h. They were then stored at 7°C in a slide box. The streptavidin-coated slides were used within two months.

### 3.4. Antibody and Microarray Slide Preparation for Bacterial Capture

The biotinylated anti-*E. coli* antibodies (and biotinylated fluorescein) were reconstituted in 60% PBS:40% glycerol (v/v) in order to prevent evaporation of the droplets and maintain a hydrated state for the capture antibodies [11,17]. Serial dilutions of biotinylated capture antibody were prepared with 60% PBS:40% glycerol, taking care to use well-mixed solutions each time.

Ten microliters of thoroughly-mixed capture antibody or biotinylated fluorescein solution were pipetted into a well of the 384-well source plate on the microarray printer. The array printing robot was controlled with SPOCLE software (TeleChem International, Inc). Contact printing, using default wash and contact settings, was conducted with one of the following five pins (TeleChem International, Inc.) that produced different spot diameters: SMP3 (100 μm spot diameter), SMP4 (135 μm), SMP6 (200 μm), SMP10 (335 μm), and SMP15 (500 μm). In order to achieve different spot diameters, the pins delivered volumes of 0.7, 1.1, 1.8, 3.9, and 7.0 nL per contact stroke, respectively. The pins were manually sonicated for 5 min in distilled H_2_O after each daily printing routine, and allowed to dry overnight before reuse. Spots were spaced 750 μm apart on each slide. Each slide was visually examined after printing to ensure that a spot was printed with each pin stroke. For slides that were going to be used without a coverslip, a hydrophobic barrier (9 mm × 10 mm) was created around the spots with a PAP pen. Within 30 min of printing completion, the slides were stored at 7°C overnight before being used the next day (approximately 18 h).

### 3.5. Growth and Enumeration of E. coli O157:H7

One milliliter of frozen stationary phase *E. coli* O157:H7 was thawed and added to 10 mL of EC broth. This was incubated at 37°C for 18 h with shaking at 160 rpm. Cultures were enumerated with a Petroff-Hausser counting chamber as described by Gehring *et al.* [20]. A 1 mL aliquot of cells was pelleted by centrifugation at 5,000 rpm for 5 min, and was resuspended in PBS and serially diluted to the desired concentrations.

### 3.6. Antibody Microarray Detection of Bacteria

Microarray slides and blocking solution were removed from refrigeration and allowed to warm briefly. In order to prevent nonspecific binding, the slides were blocked with 100 μL PBS plus 1% BSA (w/v) for 1 h at RT. The slides were then washed with continuous mixing in PBS for 3 min using a slide holder and washing jars, and were then dried with centrifugation at 2,000 rpm for 2 min. One hundred microliters of bacterial solution were then added, and each array was incubated at RT for 1 h to allow bacterial capture. Bacterial solutions consisted of either 10^6^ or 10^8-9^ cells/mL. These concentrations represented the lower end of the linear response (10^6^), and the upper end of the linear response (or saturation, 10^8-9^ [17]). During the incubation for bacterial capture, the reporter antibody solutions were prepared. Frozen aliquots of reporter antibody were thawed and diluted (typically 1:10) with PBS plus 0.5% BSA (w/v). The reporter antibody and biotinylated fluorescein were carefully light-protected during all experiments. The slides were washed 3 times (3 min each) and dried as above. Next, 100 μL reporter antibody solution was added to each slide, which was incubated for 1 h at RT. Slides were washed twice (3 min each) and dried as above, and were then scanned at the appropriate fluorescence setting (excitation: 488 nm, emission filter: 535 nm) on the laser scanner using default settings (including small/confocal pinhole autofocusing and 40 μm resolution). Note, although reactions/incubations were not carried out in a humidity-controlled environment, no significant evaporation of solvent was observed. Further details on the detection procedures are available in the figure captions.

### 3.7. Biotinylated Fluorescein Experiments

In order to determine the effectiveness of coating the microarray slides with streptavidin, and to examine washing and spot size effects, experiments with biotinylated fluorescein were conducted. Further details on the procedures described here are available in the figure captions. Experiments with biotinylated fluorescein followed procedures similar to bacterial detection. The slides were printed with biotinylated fluorescein solution as described above, and either scanned or incubated at 7°C overnight. The slides were then removed from refrigeration the next day, and then scanned, washed, and dried (as above) before being rescanned.

### 3.8. Fluorescence Microscopy of Microarray Slides

Representative microarray spots were visualized with fluorescence microscopy using procedures similar to those reported previously [21]. Briefly, the slides were placed on an inverted microscope (Nikon Diaphot, Garden City, NY). The fluorescent images that were produced with a 100 watt mercury lamp, with light traveling through a cube filter (Nikon; excitation: 470 ± 20 nm, emission: > 520 nm), were captured digitally using software (IPLab v. 3.55, Scanalytics, Inc., Rockville, MD).

### 3.9. Data Analysis

Each microarray slide, which contained at least duplicate printed spots, was considered an experimental unit. Localized background fluorescence was determined by taking random fluorescence “spot” measurements, of identical spot size (setting 15 × 15 with ScanAlyze, Dr. Michael Eisen Laboratory, University of California at Berkeley), near the array spots that were used for bacterial detection [17]. Localized background fluorescence and background fluorescent measurements (i.e., fluorescence of a spot in the absence of captured bacteria) were found to be very similar [17]. The data shown throughout indicate means ± standard deviations.

## 4. Conclusions

The properties of a 60% PBS:40% glycerol (v/v) print buffer solution, containing biotinylated capture antibodies, on a streptavidin-coated glass microarray surface were thixotropic-like and affected our microarray immunoassay results during whole cell bacterial capture and detection. During normal assaying conditions for bacterial detection, unbound biotinylated capture antibodies could be used to optimize the fluorescence signal, probably by capturing more bacteria. However, increasing the volume of print buffer, in order to make wider spots of capture antibodies (up to 500 μm), was detrimental to the immunoassay results. Capture antibody concentration of the microarray spots, method of applying solutions and cells to the microarray surface, cells available for capture, and the volume of print buffer solution used to make spots, all markedly affected the immunoassay results and should be carefully considered when developing microarray immunoassays for detection of bacterial cells.
